# Risk factors of early postoperative cardiac arrhythmia after pediatric cardiac surgery: A single-center experience

**DOI:** 10.15537/smj.2022.43.10.20220275

**Published:** 2022-10

**Authors:** Rahaf K. Alotaibi, Abdulmuti S. Saleem, Fai F. Alsharef, Zainab A. Alnemer, Yazan M. Saber, Gaser A. Abdelmohsen, Saud A. Bahaidarah

**Affiliations:** *From the Faculty of Medicine (Alotaibi, Saleem, Alsharef, Alnemer, Saber), King Abdulaziz University; from the Division of Paediatric Cardiology (Abdelmohsen, Bahaidarah), Department of Paediatrics, King Abdulaziz University Hospital, Jeddah, Kingdom of Saudi Arabia, and from the Division of Paediatric Cardiology (Abdelmohsen), Department of Paediatrics, Kasr Al Ainy School of Medicine, Cairo University, Cairo, Eygpt.*

**Keywords:** postoperative, pediatric cardiac surgery, arrhythmia, junctional ectopic tachycardia

## Abstract

**Objectives:**

To evaluate the incidence of arrhythmia in the early postoperative period and to identify its risk factors among pediatric patients following cardiac surgery at King Abdulaziz University Hospital (KAUH), Jeddah, Saudi Arabia, between 2015-2020.

**Methods:**

Out of 1242 patients, a total of 821 aged <18 years who underwent cardiac surgery were included in this retrospective cohort carried out in June 2021 at KAUH, Jeddah, Saudi Arabia. Information retrieved from the hospital medical records had patients’ demographics, types of arrhythmias, hemodynamic stability, electrolyte disturbances, cardiopulmonary bypass (CPB), and aortic cross-clamp (AXC) durations. Univariate and multivariate logistic regression analyses were used to evaluate the possible risk factors associated with postoperative arrhythmia.

**Results:**

Of the 821 patients, 140 (17.1%) developed arrhythmia postoperatively. The most common arrhythmias were junctional ectopic tachycardia (JET, 51.4%), atrioventricular block (27.1%), and supraventricular tachycardia (10%). The majority of cases occurred on the first day postoperatively (79.3%). Patients with postoperative arrhythmias had a more prolonged CPB (*p*=0.0001) and AXC (*p*=0.005) time, electrolytes disturbances (*p*=0.021), and hemodynamic instability (*p*=0.0001) than other patients.

**Conclusion:**

Postoperative arrhythmia, especially JET, is common after pediatric cardiac surgery. Prolonged cardiopulmonary bypass, prolonged aortic cross-clamping, electrolytes disturbances, and hemodynamic instability are possible risk factors for postoperative cardiac arrhythmias.

**A**rrhythmia is a disturbance in cardiac rhythm or rate.^1^ It can be a transient phenomenon provoked by irritation of the conductive system or persistent caused by scarring and damage to the myocardium.^2,3^ **A**rrhythmias can be divided according to different classifications, mainly into 2 types: bradyarrhythmia (slow rhythm) and tachyarrhythmia (rapid rhythm).^4^ It is a well-known complication of cardiac procedures among pediatric patients, with a reported incidence of 7.5-48% and a mortality rate of 9.1%.^1,5-12^ Moreover, a significant number of these arrhythmias are associated with certain surgeries, most commonly with surgical repair of ventricular septal defect (VSD), atrial septal defect (ASD), and tetralogy of Fallot (TOF).^2^ Multiple risk factors have been reported to increase the possibility of developing arrhythmia, including younger age and lower body weight; more prolonged cardiopulmonary bypass (CPB) duration and aortic cross-clamp (AXC) time; and higher lactic acid levels.^13^ Most arrhythmias are temporary or self-limiting and resolve with adequate therapy, such as correcting electrolytes or metabolic disturbances. Nevertheless, some arrhythmias, particularly bradyarrhythmia, may necessitate permanent pacemakers.^14^ Other types of arrhythmias can be managed by cardioversion or medications, such as amiodarone.^15^

A prospective study carried out in Poland, including 402 pediatric patients with congenital heart diseases, revealed that, postoperatively, 57 (14.2%) out of 402 patients suffered from arrhythmia. This study showed that junctional ectopic tachycardia (JET) was the most common type of arrhythmia, followed by supraventricular tachycadia (SVT), and arteriovenous (AV) block.^15^ Similarly, a study published in the United States of America in 2015 reported that JET was the most prevalent arrhythmia, accounting for 7.5% of cases.^11^ In 2019, a retrospective study reported that surgeries involving manipulation of the conduction system or requiring prominent dissection were associated with AV block, with a prevalence of 5.2%.^14^ Furthermore, a cohort study in 2021 showed that the frequency and diagnosis of arrhythmias might differ depending on the type of cardiac procedure.^16^ A study in Switzerland found that various variables could cause or aggravate postoperative arrhythmias. These variables included pre-existing myocardial compromise due to cardiac defects, complex procedures with extensive scars and suture lines, consequences of myocardial ischemia, postoperative electrolyte disturbances, and increased adrenergic tone or catecholamine stimulation.^17^ In another study in 2019 in India, risk factors were found to include: I) young age and low body weight at surgery; II) longer CPB duration and AXC time; and III) temperature disturbances, use of deep hypothermia, and circulatory arrest.^2^

Cardiac arrhythmia is a common cause of sudden death worldwide. Therefore, early arrhythmia detection is crucial to ensure prompt management and prevent serious complications, which is also valuable for improving total patient care. However, limited studies have evaluated the incidence rate and risk factors for developing arrhythmia in children following cardiac surgery in Saudi Arabia. Accordingly, this study aimed to assess the prevalence of arrhythmia and its possible risk factors among pediatric patients who underwent cardiac procedures at King Abdulaziz University Hospital (KAUH), Jeddah, Saudi Arabia, between 2015-2020.

## Methods

This retrospective record review study was carried out in the Department of Pediatrics at KAUH, Jeddah, Saudi Arabia, in June 2021. This study was approved by the Institutional Review Board of KAUH (approval number: 39-21). The requirement for informed consent was waived due to the study’s retrospective nature. Our inclusion criteria included: male and female patients under 18 years who underwent cardiac surgery. Patients with known comorbidities, a history of arrhythmia preceding surgery, or children who died after surgery were excluded from the study. The data were collected in June 2021 by 5th-year medical students using data collection sheets to review the records of patients who underwent cardiac surgeries between January 2015 and December 2020. The extracted data comprised the patients’ demographic characteristics: age, gender, height, weight, and nationality. In addition to clinical diagnosis and history of arrhythmia before surgery, operative data included type of opertion, CPB duration, AXC time, hemodynamic instability, and abnormal electrolyte levels. Hemodynamic instability is the presence of hypotension for age, oliguria/anuria, metabolic acidosis, and high lactate levels above the normal reference range for age. Normal electrolyte levels in this study were defined as serum potassium level of 3.5-5 mmol/L, sodium of 136-145 mmol/L, calcium of 2.2-2.6 mmol/L, and magnesium of 0.66-1.07 mmol/L.^18^ Data on abnormal oxygen saturation levels and body temperature were collected. This study also included data on newly developed arrhythmia and its types (SVT, AV block II, AV block III, JET, atrial premature complexes, ventricular premature complexes, junctional escape rhythm, ventricular tachycardia, and atrial fibrillation). Data on the time of arrhythmia development (on the postoperative day: one, 2, or 3) and its duration (<24 hours, 24-48 hours, or >48 hours) were also collected. Data on type of interventions for arrhythmia, whether spontaneously relieved or necessitated electrolyte correction, electric cardioversion, pacemaker insertion (temporary or permanent), or medications (such as amiodarone, quinidine, procainamide, lidocaine, propranolol, diltiazem, or digitalis) were extracted. Microsoft Excel 2020 was used for data entry.

### Statistical analysis

Data analysis was carried out using the Statistical Package for the Social Sciences, version 26.0 (IBM Corp., Armonk, NY, USA). Numeric data were tested for normality using Kolmogorov-Smirnov and Shapiro-Wilk tests, and as most of our data were non-normally distributed, we expressed numeric data as the median and interquartile range (25^th^-75^th^ percentile). Nominal variables were presented as numbers or numbers and percentages. Comparisons between groups were tested using Chi-square tests for categorical variables and non-parametric Mann-Whitney-U test for numeric variables. Univariate logistic regression analysis was used for evaluating the predictors of postoperative arrhythmias then variables of statistical significance were incorporated into a multivariable stepwise logistic regression model. A *p*-value of <0.05 was considered significant.

## Results

A total of 1242 patients underwent pediatric cardiac surgeries between 2015-2020, of which 821 fulfilled the study’s inclusion criteria. The recruited patients comprised males (54.6%) and females (45.4%). Approximately half of the patients (51%) were infants (up to one year), and the median weight of patients was 7.4 kg. Table 1 illustrates patients’ demographic and the clinical data of patient groups.

**Table 1:** - Demographic and clinical characteristics of studied patients (N=821).

Variables	n (%)
***Gender***	
Male Female	448 (54.6) 373 (45.4)
***Nationality***	
Non-Saudi Saudi	706 (86.0) 115 (14.0)
***Age groups***	
<1 month 1-6 months >6-12 months >1-6 years >6-12 years >12-18 years	45 (5.5) 184 (22.4) 188 (22.9) 257 (31.3) 125 (15.2) 22 (2.7)
Height (cm), median (IQR)	71 (57-97)
Weight (Kg), median (IQR)	7.4 (4.2-13)
BMI, median (IQR)	14.15 (12.5-16)
Hemodynamically instability	171 (20.8)
***Presence of electrolyte disturbances***	743 (90.5)
Abnormal serum sodium level Abnormal serum potassium level Abnormal serum calcium level Abnormal serum magnesium level	525 (70.7) 311 (41.9) 395 (53.2) 635 (85.5)
Hypoxia	272 (33.1)
***Abnormal body temperature***	
Hyperthermia Hypothermia	35 (67.3) 17 (32.7)
CPB time (minutes), median (IQR)	63 (47-84)
AXC time (minutes), median (IQR)	47 (32-63)

Values are presented as a number and precentage (%).

BMI: body mass index, CPB: cardiopulmonary bypass,

AXC: aortic cross-clamp, IQR: interquartile range (25th-75th percentile)

Cardiac operations were carried out under CPB in 777 patients, and only 44 procedures were carried out off-pump. The median duration of CPB was 63 minutes, whereas the median AXC time was 47 minutes. Hemodynamic instability was observed in 20.8%, and electrolyte disturbances in 90.5%, as shown in Table 1.

In this cohort, the most frequent operations were VSD closure (17.8%), Fallot repair (13.5%), and single ventricle palliation (17.9%). Types of procedures carried out were summarized in Appedix 1.

Arrhythmia was reported in 140 (17.1%) out of 821 cardiac surgeries. Moreover, 137 arrhythmic cases occurred in open heart procedure, while only 3 cases were of the pump. Most of the arrhythmias occurred on the first day post-operation (79.3%) and lasted less than 24 hours in 49%. The most frequent type of arrhythmia was JET (51.4%), followed by AV block III (27.1%), and SVT (10%). Table 2 summarizes various types of arrhythmias reported in addition to the onset and duration of each type.

**Table 2:** - Arrhythmia types, duration, and onset in patients who had postoperative arrhythmias.

Type of arrhythmia	Frequency of arrhythmia	Duration of arrhythmia, n			Postoperative day of onset, n			
		<24 hours	24-48 hours	>48 hours	1^st^ day	2^nd^ day	3^rd^ day	After 3^rd^ day
SVT	14 (10.0)	7	3	4	7	2	0	5
PVC	4 (2.9)	0	3	1	1	0	1	2
JET	72 (51.4)	39	12	21	58	9	1	4
AV block III	38 (27.1)	14	3	21	34	1	2	1
AV block II	1 (0.7)	1	0	0	1	0	0	0
AV block I	2 (1.4)	1	1	0	1	0	0	1
V-tach	2 (1.4)	2	0	0	2	0	0	0
V-fib	2 (1.4)	2	0	0	2	0	0	0
Atrial fib	3 (2.1)	2	1	0	3	0	0	0
Other	2 (1.4)	1	0	1	2	0	0	0
Total	140 (100)	69	23	48	11	12	4	13

Values are presented as a number and precentage (%). SVT: supraventricular tachycardia, PVC: premature ventricular complex, JET: junctional ectopic tachycardia, AV: atrioventricular, V-tach: ventricular tachycardia, V-fib: ventricular fibrillation, atrial fib: atrial fibrillation

Specific surgical operations were associated with a high incidence of arrhythmia. For instance, repair of VSD accounted for 18.6% of arrhythmia cases (mainly third-degree AV block). Moreover, TOF and atrioventricular septal defect (AVSD) repairs were equally related to 15% of all arrhythmic cases, predominantly manifested as JET.

**Figure 1:**
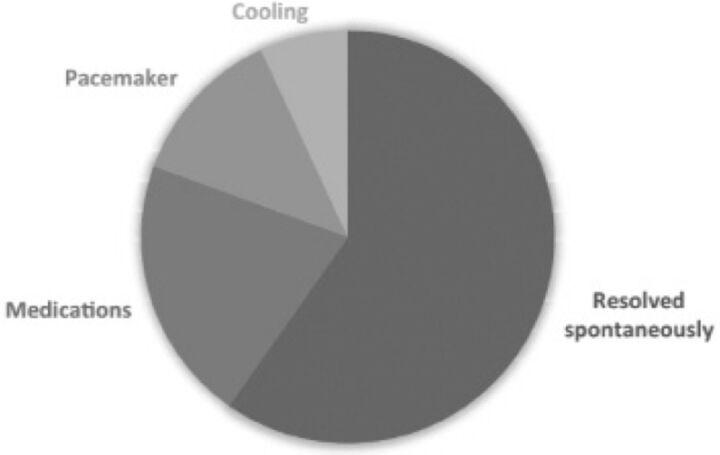
- Management of junctional ectopic tachycardia

Of the 72 patients with JET, arrhythmia resolved spontaneously in 59.7%, 20.8% responded to medications such as amiodarone, 12.5% required placement of temporary pacemakers for overdrive pacing, and 6.9% of these cases were treated with cooling as illustrated in Figure 1. Most patients with JET had abnormal electrolyte levels (95.8%), and 30.6% were hemodynamically unstable. Cases of JET were primarily detected on postoperative day one (80.6%), and arrhythmia was terminated within the first 24 hours in 54.2% of the patients and persisted for more than 48 hours in 29.2%.

A total of 38 cases developed third-degree AV block, 89.5% were reported within postoperative day one, and it lasted for more than 48 hours in 55.3% of the cases. These patients had a temporary pacemaker, while permanent pacemakers were indicated in 6 patients with persistent 3^rd^ degree AV block beyond the 10^th^ day after cardiac surgery. Electrolyte disturbances were reported in all AV block cases. However, only 7.9% of the cases required electrolytes correction. Hemodynamic instability was observed in 34.2% of the patients.

Among the 14 patients with SVT, 57.1% were hemodynamically unstable, and 92.9% had electrolyte imbalance. Amiodarone and propranolol were administered in 85.7% of the cases. Furthermore, 28.6% of these cases needed correction of electrolytes, and none required pacemaker insertion. Moreover, half of the patients with SVT were detected on postoperative day one, and arrhythmia lasted for less than 24 hours.

In this cohort, patients with postoperative arrhythmias had a more prolonged CPB (*p*=0.0001) and AXC (*p*=0.005) time, electrolyte disturbances (*p*=0.021), and hemodynamic instability (*p*=0.0001) than other patients, as illustrated in Table 3. Interestingly patients with postoperative arrhythmias had lower magnesium levels than others (*p*=0.025). Prolonged CPB and AXC times, electrolyte disturbances, and hemodynamic instability were independent risk factors for developing postoperative arrhythmias in univariate logistic regression analysis. Hemodynamic instability and long CPB time were the most critical risk factors for developing postoperative arrhythmias after stepwise logistic regression analysis, as shown in Table 4.

**Table 3:** - Comparison between patients having postoperative arrhythmias with no arrhythmia patients after cardiac surgery.

Variables	Arrhythmia group (n=140)	No arrhythmia groups (n=681)	*P*-values
***Age groups***			
<1 month 1-6 months >6-12 months >1-6 years >6-12 years >12-18 years	9 (6.4) 42 (30.0) 34 (24.3) 39 (27.9) 13 (9.3) 3 (2.1)	36 (5.3) 142 (21.0) 154 (22.6) 218 (32.0) 112 (16.4) 19 (2.8)	0.091
Weight (Kg), median (IQR)	6.6 (4-12.8)	7.4 (4.4-13)	0.351
CPB time (minutes), median (IQR)	77 (57-99.5)	60 (45-81)	0.0001*
AXC time (minutes), median (IQR)	54 (38-70)	45 (32-62)	0.005*
Hemodynamic instability	49 (35.0)	122 (18.0)	0.0001*
Abnormal body temperature	9 (6.4)	43 (6.3)	0.960
Hypoxemia	50 (35.7)	222 (32.6)	0.510
Electrolytes imbalance	134 (95.7)	609 (89.4)	0.021*

Values are presented as a number and precentage (%). *Statistically significant. CPB: cardiopulmonary bypass, AXC: aortic cross-clamp, IQR: interquartile range (25th-75th percentile)

## Discussion

The primary objectives of this study were to evaluate the incidence of arrhythmia and secondarily, determine potential risk factors in the pediatric age group after undergoing cardiac procedures at KAUH, Jeddah, Saudi Arabia, between 2015-2020. In this record review, the incidence rate of arrhythmia encountered by children following cardiac operations was 17.1%. However, unlike our results, this incidence may reach up to 48%.^9^ Moreover, previous studies by Jain et al,^14^ showed an incidence rate of 14.4%, Pfammatter et al,^8^ showed an incidence rate of 27%, and Valsangiacomo et al,^7^ showed an incidence rate of 48%. The disparity in the majority of arrhythmia between studies may be due to differences in the definition of arrhythmia and the selection of a more sensitive method to detect it. For instance, Grosse-Wortmann et al^10^ used the Holter monitor, a highly sensitive method for detecting arrhythmias compared to the regular bedside monitor used in this study; they recorded an incidence rate of 59% in neonates and 79% in older children. As previously mentioned, different definitions of arrhythmia may contribute to percentage variations between studies. Yildirim et al^1^ considered “persistent arrhythmias lasting for more than 30 seconds” as a definition, whereas Delaney et al^9^ defined arrhythmias as events that required intervention.

**Table 4:** - Predictors for developing postoperative arrhythmias after cardiac surgery.

Variables	Univariate logistic regression analysis		Multivariate logistic regression analysis	
	OR (95% CI)	*P*-values	OR (95% CI)	*P*-values
AXC time	1.011 (1.003-1.019)	0.009*	0.995 (0.979-1.010)	0.500
Associated electrolyte disturbances	2.640 (1.125-6.200)	0.026*	2.310 (0.804-6.634)	0.120
Associated hemodynamic instability	2.467 (1.656-3.766)	0.0001*	1.772 (1.104-2.884)	0.018*
CPB duration	1.014 (1.008-1.019)	0.0001*	1.014 (1.002-1.027)	0.024*

* Statistically significant. AXC: aortic cross-clamping, CPB: cardiopulmonary bypass, OR: odds ratio, CI: confidence interval

Junctional ectopic tachycardia was the most common type of arrhythmia shown postoperatively in the current study (51.4% of arrhythmia patients). Most arrhythmias occur on first day postoperatively and last for less than 24 hours. Similarly, JET represented 45.2% of the arrhythmias reported by Öztürk et al.^16^ Other authors reported much lower percentages of JET, ranging between 2-11%.^15,19^ Previous reports showed the occurrence of JET mainly following AVSD and TOF repairs.^20-22^

Hemodynamic instability and electrolyte disturbances may play a role in developing postoperative arrhythmia. In this cohort, most cases of JET had abnormal electrolyte levels. Medications were required in 15 patients and overdrive pacing were required in 9 patients using temporary pacemakers. In a study in Poland, overdrive pacing was the only intervention used to control JET.^15^

In the present study, AV block III accounted for 27.1% of all arrhythmia cases. Repair of VSD was the principal operation complicated by AV block III, followed by AVSD repair. Complete heart block is a common complication of procedures with maximum manipulation of the heart conducting system, operations requiring significant surgical dissection, and those complex. Advances in surgical techniques and further understanding of the anatomy of the heart conduction system have decreased the incidence of AV block III from 25% to approximately 5%.^23,24^ This arrhythmia tended to persist for longer durations, necessitating cardiac pacing. In a study carried out in India in 2019, a pacemaker was used as an intervention in all cases with AV block III; pacemakers were permanently placed in 3 patients while the other 23 were on a temporary pacemaker until they recovered.^14^

Similarly, in the current study, AV block III was mainly managed conservatively using a temporary pacemaker, and a small number of patients had persistent heart block that required permanent pacing. Unlike our study, AV block III was the most prevalent type of arrhythmia in the study by Jain et al.^14^ Several reasons can explain this; first, different types of operation may result in the development of specific types of arrhythmia, such as AV block III arising from surgeries that involve dissection of deep structures irritating the heart conduction system; and second, the surgical technique used may be a contributing factor and should be considered.

The current study noted SVT in 14 patients, accounting for 10% of total events. Conversely, other studies reported that SVT was one of the most frequent arrhythmias observed in postoperative cardiac surgery units, with a prevalence ranging from 1-30%.^1,7,9,15^ Moreover, studies in Turkey found that SVT was related to ASD repair with partial anomalous pulmonary venous return and Rastelli surgery.^1,2^ However, AVSD, TGA correction, and Glenn shunt were the most prevalent operations related to SVT, as observed in our study and another report.^22^

More than half of the patients experienced hemodynamic instability and electrolyte imbalance. In a study in India, of 8 cases with SVT, 6 (75%) developed it within 24 hours after surgery, and 5 (62.5%) experienced recurrent episodes, necessitating the use of amiodarone. Only one case with hemodynamic instability necessitated synchronized cardioversion and amiodarone treatment.^14^ In comparison to our intervention, almost all patients received amiodarone or propranolol. Since amiodarone is the drug of choice for treating SVTs in many studies, it was chosen based on its well-documented antiarrhythmic potency, limited hemodynamic side effects, and good tolerance in children.^8,22,25,26^ The remaining 2 (14.3%) patients recovered spontaneously.

Irrespective of the type of arrhythmia, most were seen on postoperative day one with a percentage of 79.3%. A study in Indonesia revealed that 45 out of 191 patients developed an arrhythmia, 31 of whom experienced arrhythmia on their first day following surgery.^27^ This can be justified by the fact that most studies primarily focused on the early days after surgery, in which a few arrhythmias that may develop later on might have been missed. Another explanation is that hemodynamic instability during surgery may give rise to arrhythmia in this period. This is in line with the current study, as most hemodynamic-related arrhythmias were observed within the first 24 hours following surgery.

Numerous studies have identified several risk factors for postoperative arrhythmia development, including patient-related factors such as younger age and lower body weight during operation.^7,9,15^ However, these factors, along with oxygen saturation and body temperature abnormalities, were insignificantly linked to arrhythmia. Nonetheless, electrolyte disturbance and hemodynamic instability were significantly associated with arrhythmia.

In the present study, hemodynamic instability, electrolytes disturbance, and prolonged CBP and AXC times were indefinable risk factors for developing postoperative arrhythmia, especially the hemodynamic instability and long CBP time like previous reports Öztürk et al^16^ and Jain et al.^14^ These results were consistent with our findings reported in the present study. This might be due to cardioplegia, a part of the CPB process; the heart becomes perfused with a solution to induce electromechanical arrest, which can develop arrhythmia.^28^ Other risk factors included higher Aristotle basic scores, which measure the degree of complexity of surgery carried out. In addition, a higher grade of the Risk Adjustment for Congenital Heart Surgery-1 that predicts outcomes of cardiac operation in the high-risk population was a risk factor according to Öztürk et al^16^ and Rękawek et al.^15^

### Study limitations

Primarily, the retrospective nature of the analysis has limited our ability to observe the patients and follow them up. Additionally, it was carried out in a single center only. Second, poor documentation of the hospital system has led to the exclusion of a few files. Another limitation was detecting arrhythmias, in which a regular bedside monitor was used, and a few cases of rhythmic disturbances might have been missed. In Saudi Arabia, further prospective studies are recommended to enable a closer follow-up of patients following surgery. Moreover, using more sensitive monitors would facilitate more accurate detection of arrhythmias.

In conclusion, postoperative arrhythmia is a significant complication that increases mortality and morbidity rates. In this study, the incidence of arrhythmia was 17.1%, with JET being the most common type, followed by AV block III, and SVT. Prolonged CPB and AXC duration, electrolyte imbalance, and hemodynamic instability were possible risk factors for developing postoperative arrhythmia. Even though most of the arrhythmias resolved spontaneously, some were persistent and life-threatening and needed further interventions.

## Appendix

**Appendix 1:** - Types of cardiac operations carried out.

Type of operations	n	%
Atrioventricular septal defect repair	65	7.9
Atrial septal defect repair	30	3.7
Ventricular septal defect repair	146	17.8
Teratology of Fallot repair	111	13.5
Right ventricle outflow tract resection	3	0.4
Double outlet right ventricle repair	16	1.9
Transposition of the great arteries repair	66	8.0
***Pulmonary vein surgery***		
Total anomalous pulmonary venous drainage repair Pulmonary artery pulmonary venous drainage-atrial septal defect repair Pulmonary vein stenosis repair	18 1 3	2.2 0.1 0.4
***Aortic valve/arch repair surgery***		
Interrupted aortic arch repair Coarctation repair Aortic arch repair Patch aorto-plasty Subaortic posterior annulus patch Aortic valvuloplasty Aortic valve replacement Left ventricular outflow tract fibro-myectomy Ross AVR	6 31 7 4 1 9 3 21 1	0.7 3.8 0.9 0.5 0.1 1.1 0.4 2.6 0.1
***Pulmonary artery surgery***		
Patent ductus ligation Pulmonary artery plasty	14 4	1.7 0.5
***Mitral valve surgery***		
Mitral valvuloplasty Mitral annuloplasty Mitral valve replacement Supravalvular mitral ring resection Cor-triatriatum repair	8 11 8 1 2	1.0 1.3 1.0 0.1 0.2
***Pulmonary valve surgery***		
Pulmonary valvuloplasty Pulmonary valvotomy Pulmonary valve replacement	1 2 4	0.1 0.2 0.5
Tricuspid valve surgery	3	0.4
***Rastelli operation***	5	0.6
Conduit right ventricle to the pulmonary artery - valved	9	1.1
***Single ventricle palliation***		
Glenn shunt Norwood operation Fontan operation Damus-Kaye-Stansel connection	78 17 48 4	9.5 2.1 5.8 0.5
Central Shunt with graft	3	0.4
Coronary repair anomalous left coronary artery from the pulmonary artery	1	0.1
Truncus arteriosus repair	9	1.1
Blalock-taussig	8	1.0
***Other***	7	0.8
A-P window repair Atrial septectomy Unifocalization of pulmonary atresia Ventricular septal defect enlargement Vascular ring repair Pulmonary artery banding-MPA Pulmonary artery de-banding Extracorporeal membrane oxygenation Pacemaker-primary implant	3 4 6 1 2 7 1 6 2	0.4 0.5 0.7 0.1 0.2 0.9 0.1 0.7 0.2
Total	821	100.0

Values are presented as a number and precentage (%).
